# Prevalence and Correlates of the Belief That Electronic Cigarettes are a Lot Less Harmful Than Conventional Cigarettes Under the Different Regulatory Environments of Australia and the United Kingdom

**DOI:** 10.1093/ntr/ntw137

**Published:** 2016-05-17

**Authors:** Hua-Hie Yong, Ron Borland, James Balmford, Sara C. Hitchman, K. Michael Cummings, Pete Driezen, Mary E. Thompson

**Affiliations:** ^1^Nigel Gray Fellowship Group, Cancer Council Victoria, Melbourne, Australia;; ^2^Department of Addictions, Institute of Psychiatry, Psychology, and Neuroscience, King’s College London, London, United Kingdom;; ^3^UK Centre for Tobacco and Alcohol Studies (UKCTAS), United Kingdom;; ^4^Department of Psychiatry and Behavioral Sciences, Medical University of South Carolina, Charleston, SC;; ^5^Department of Psychology, University of Waterloo, Waterloo, ON, Canada;; ^6^Department of Statistics and Actuarial Science, University of Waterloo, Waterloo, ON, Canada

## Abstract

**Introduction:**

The rapid rise in electronic cigarettes (ECs) globally has stimulated much debate about the relative risk and public health impact of this new emerging product category as compared to conventional cigarettes. The sale and marketing of ECs containing nicotine are banned in many countries (eg, Australia) but are allowed in others (eg, United Kingdom). This study examined prevalence and correlates of the belief that ECs are a lot less harmful than conventional cigarettes under the different regulatory environments in Australia (ie, more restrictive) and the United Kingdom (ie, less restrictive).

**Methods:**

Australian and UK data from the 2013 survey of the International Tobacco Control Four-Country project were analyzed.

**Results:**

More UK than Australian respondents (58.5% vs. 35.2%) believed that ECs are a lot less harmful than conventional cigarettes but more respondents in Australia than in the United Kingdom selected “Don’t Know” (36.5% vs. 17.1%). The proportion that responded “A little less, equally or more harmful” did not differ between countries. Correlates of the belief that ECs are “A lot less harmful” differed between countries, while correlates of “Don’t Know” response did not differ.

**Conclusions:**

Consistent with the less restrictive regulatory environment affecting the sale and marketing of ECs, smokers and recent ex-smokers in the United Kingdom were more likely to believe ECs were less harmful relative to conventional cigarettes compared to those in Australia.

**Implications:**

*What this study adds:* Among smokers and ex-smokers, this study found that the belief that ECs are (a lot) less harmful than conventional cigarettes was considerably higher in the United Kingdom than in Australia in 2013. The finding is consistent with the less restrictive regulatory environment for ECs in the United Kingdom, suggesting that the regulatory framework for ECs adopted by a country can affect smokers’ perceptions about the relative harmfulness of ECs, the group that stands to gain the most from having an accurate belief about the relative harms of ECs.

## Introduction

Use of electronic cigarettes (ECs) has risen rapidly in recent years^1–3^ and this has fuelled debate in the public health community on how to regulate ECs. In many countries, there are currently no specific laws that directly apply to ECs. This is the case in Australia where a number of existing laws relating to poisons, therapeutic goods and tobacco control apply to ECs in some circumstances. ECs containing nicotine cannot be legally sold without a permit or a doctor’s prescription in Australia. However, non-nicotine containing ECs can be sold and used lawfully. However, in several states ECs are effectively banned under legislation prohibiting products that look like cigarettes.^4^ Advertising of ECs is also effectively banned, although some exposure to advertising may still occur in Australia via the internet.

By contrast, in countries like the United Kingdom there are few restrictions on the marketing and sale of ECs. In May 2014 the EU tobacco products directive covering ECs came into force, which introduced controls on product characteristics, nicotine content, and labeling, and prohibited some types of EC marketing when sold as consumer goods. However, EU countries have until mid-2016 to implement the tobacco products directive. The UK Medicines and Healthcare Products Regulatory Agency (MHRA) has also encouraged EC manufacturers to apply for medicinal licences for their products to be approved as for nicotine replacement therapies; however few manufacturers have taken advantage of this opportunity with only one EC to date having been registered and approved,^5^ and no EC products are currently available for sale as licensed nicotine therapeutics. England is the first part of the United Kingdom, and also the first place in the world, to promote licensed nicotine-containing products, including any licensed ECs, for harm reduction in line with guidance from the National Institute for Health Care Excellence (NICE).^6^ At the time this study was conducted, marketing of ECs in the United Kingdom was permitted, but under general advertising rules several EC ads had been banned for making misleading claims.

The long-term health risks of EC use are unknown at this time, but compared with conventional cigarettes, ECs are almost certainly less harmful to users and bystanders.^7–9^ While the use of ECs is strongly predicted by risk perceptions in relation to conventional cigarettes,^2,10,11^ the extent to which different regulatory environments might affect smokers’ beliefs about EC relative harmfulness is unknown. We would expect ECs to be considered safer than conventional cigarettes when the regulatory environment is less restrictive in regards to marketing of ECs, as in the United Kingdom relative to Australia. In the United Kingdom, the marketing of ECs in the form of implicit and explicit claims via advertising of reduced harm for EC products and also by public endorsements of ECs from multiple respected health organizations (eg, Action on Smoking and Health,^12,13^ Royal College of Physicians^14^), are likely to have generated a more favorable public perception about EC risks and/or relative risks. Nevertheless, there are also public dis-endorsements (eg, British Medical Association^15^) and a number of popular media outlets have published articles warning that ECs and their emitted aerosols are dangerous.^16^ By contrast, the restrictions on EC marketing and retail sales in Australia combined with mainly public dis-endorsements of ECs from prominent health organizations (eg, Cancer Councils, National Health Foundation) and prominent tobacco control advocates are likely to create a perception that the products are harmful and thus, increase the perceived risk or uncertainties about the products.

This study (1) examines prevalence and correlates of beliefs about the relative harmfulness of ECs and conventional cigarettes among smokers and ex-smokers in Australia and the United Kingdom, and (2) determines whether these differed across the two countries. Insights gained from this comparative study are likely to be useful for informing other countries on policy with respect to ECs.

## Methods

### Sample and Data Collection Procedures

Data analyzed were from the Australian and United Kingdom (collected between February to May and February to September, 2013, respectively) arm of the International Tobacco Control Four-Country (ITC) project, a longitudinal cohort study of adult smokers followed up approximately yearly with replenishment of cases lost to the study (study design and sampling frames have been described elsewhere^17,18^). The Australian sample had significantly more participants who were younger, of higher income and more addicted than the UK sample, but the two samples were otherwise similar (Supplementary Table 1).

### Measures

EC awareness and trial were assessed using the questions “Have you ever heard of electronic cigarettes or e-cigarettes?” and “Have you ever tried an electronic cigarette?”, both answered Yes/No. Those who had tried were asked “How often, if at all, do you currently use an electronic cigarette?” with the response options “Daily, Less than daily, Less than weekly, Less than monthly or Not at all”. All participants aware of ECs were asked whether or not they thought ECs were more, less or equally harmful as regular cigarettes, with “Don’t Know” an acceptable response. Those who indicated that ECs were less harmful were followed up with the question: “Are they a little or a lot less harmful than regular cigarettes?” An ordinal measure of perceived harmfulness of ECs relative to conventional cigarettes was derived (a dichotomized version was used for regression analysis where “A lot less harmful” vs. otherwise was also explored). Respondents were also asked about noticing EC adverts in the last 6 months along with standard sociodemographics and smoking-related variables.

### Data Analysis

Prevalence estimates and 95% confidence intervals were computed for EC relative harmfulness beliefs among both smokers and ex-smokers stratified by country. Correlates of the belief that ECs are (a lot) less harmful were examined using multiple logistic regression models and tested for country differences. Additional analyses were conducted to explore correlates of answering “Don’t Know” to the question of whether ECs are less harmful than conventional cigarettes. All analyses were conducted using Stata v12.1.

## Results

### EC Risk Perceptions in Australia and the United Kingdom

Significantly more UK respondents than Australian respondents believed that ECs are a lot less harmful than conventional cigarettes (59% vs. 35%, adjusted odds ratio [*AOR*] = 0.50, *P* < .001; see Table 1). Response patterns were otherwise similar, with the exception of “Don’t Know” responses. More Australian respondents than those in the United Kingdom answered “Don’t Know” when asked about the relative harmfulness of ECs (37% vs. 17%, *AOR* = 2.34, *P* < .001).

**Table 1.** Prevalence Estimates of Relative Harmfulness Belief About Electronic Cigarettes (ECs) Among Smokers and Ex-Smokers Who Were Aware of ECs (Australia vs. the United Kingdom, 2013 Survey of the International Tobacco Control Four-Country Project)

Belief ECs as compared to conventional cigarettes	% (95% CI)			
	Smokers	Quit ≤ 12 months	Quit > 12 months	Total
Australia				
*N*	695	73	148	916
A lot less harmful	36.0 (31.2–41.2)	33.1 (21.3–47.5)	32.6 (23.4–43.5)	35.2 (31.1–39.6)
Unsure how much less harmful	4.2 (2.6–6.7)	6.2 (2.5–14.6)	4.8 (2.4–9.1)	4.5 (3.1–6.4)
A little less harmful	15.7 (11.3–21.4)	10.8 (4.5–23.9)	9.8 (4.8–18.9)	14.4 (10.8–18.9)
Don’t know	36.3 (31.4–41.4)	41.8 (26.9–58.3)	34.9 (25.6–45.5)	36.5 (32.2–40.9)
Equally harmful	7.3 (5.2–10.2)	8.0 (3.4–17.9)	17.8 (10.9–27.6)	9.1 (7.0–11.8)
More harmful	0.4 (0.2–1.1)	0	0.2 (0.0–1.5)	0.4 (0.1–0.9)
United Kingdom				
*N*	987	73	209	1269
A lot less harmful	57.6 (53.7–61.4)	72.4 (58.1–83.2)	57.2 (48.0–65.9)	58.5 (55.0–61.9)
Unsure how much less harmful	3.3 (2.3–4.7)	1.7 (0.3–7.9)	3.2 (1.5–6.5)	3.2 (2.3–4.3)
A little less harmful	11.8 (9.5–14.6)	9.1 (3.3–22.9)	16.7 (11.1–24.3)	12.4 (10.3–14.9)
Don’t know	17.5 (14.8–20.5)	13.7 (7.4–23.9)	17.0 (12.1–23.4)	17.1 (14.8–19.8)
Equally harmful	8.3 (6.5–10.6)	2.8 (0.9–8.8)	5.6 (2.9–10.6)	7.5 (6.0–9.4)
More harmful	1.6 (0.8–3.1)	0.3 (0.0–2.4)	0.3 (0.0–2.2)	1.3 (0.7–2.5)

CI = confidence interval. Estimates are based on weighted data.

### Correlates of EC Risk Perceptions in Australia and the United Kingdom

Table 2 presents correlates of the belief that ECs are a lot less harmful than conventional cigarettes (correlates of ECs being less harmful are similar, results not shown). Overall, this belief was more likely to be endorsed by respondents from the United Kingdom. In both countries, this belief was also more likely to be endorsed by those from an English-speaking background (effect significant only in Australia), those who had tried ECs before or were current users, and those who had noticed EC ads on the internet (significant only in the United Kingdom), but no effect was found for in-store advertising. Additional significant effects found in the United Kingdom but not in Australia included younger age and higher household income, both associated with greater odds of believing ECs to be a lot less harmful. Gender, education and survey mode showed differential effects across countries (significant by-country interaction: *P* = .024, .045 and .010, respectively). Australian respondents who were female and had been surveyed by phone were more likely to believe ECs to be a lot less harmful, whereas in the United Kingdom no gender or survey mode effects were found. Education was not a significant independent predictor in either country, but there were trends for higher education in Australia and lower education in the United Kingdom to be associated with believing they were less harmful.

**Table 2.** Logistic Regression Results Showing Correlates of the Belief That ECs are “A Lot Less Harmful,” and “Don’t Know” Responses, in the United Kingdom and Australia, 2013 Survey of the International Tobacco Control Four-Country Project

Variables	Believed that ECs are a lot less harmful vs. otherwise			Didn’t know whether ECs are less harmful vs. otherwise		
	*AOR* (95% CI)			*AOR* (95% CI)		
	Combined: *N* = 2105	United Kingdom: *N* = 1215	Australia: *N* = 890	Combined: *N* = 2105	United Kingdom: *N* = 1215	Australia: *N* = 890
Country						
United Kingdom	Ref	—	—	Ref	—	—
Australia	0.50 (0.41–0.61)***	—	—	2.34 (1.87–2.94)***	—	—
EC use						
Never	Ref	Ref	Ref	Ref	Ref	Ref
Tried before	1.77 (1.40–2.24)***	1.91 (1.41–2.60)***	1.72 (1.18–2.50)**	0.47 (0.35–0.62)***	0.38 (0.25–0.59)***	0.52 (0.34–0.78)**
Current use	2.13 (1.63–2.78)***	2.05 (1.48–2.83)***	2.54 (1.56–4.13)***	0.32 (0.22–0.46)***	0.31 (0.19–0.49)***	0.32 (0.18–0.57)***
Noticed EC ads on the internet						
No	Ref	Ref	Ref	Ref	Ref	Ref
Yes	1.38 (1.11–1.73)**	1.33 (1.01–1.75)*	1.44 (0.98–2.11)	0.61 (0.47–0.81)**	0.63 (0.43–0.92)*	0.62 (0.40–0.95)*
Noticed EC ads inside stores						
No	Ref	Ref	Ref	Ref	Ref	Ref
Yes	1.17 (0.94–1.47)	1.22 (0.95–1.57)	1.09 (0.63–1.89)	0.80 (0.60–1.07)	0.75 (0.54–1.05)	0.91 (0.49–1.66)
Smoking status						
Smokers	Ref	Ref	Ref	Ref	Ref	Ref
Quit ≤ 12 months	1.26 (0.84–1.89)	1.18 (0.67–2.09)	1.43 (0.79–1.59)	0.89 (0.56–1.40)	1.22 (0.60–2.49)	0.68 (0.37–1.25)
Quit > 12 months	1.10 (0.80–1.51)	1.15 (0.76–1.74)	1.03 (0.62–1.73)	0.64 (0.46–0.91)*	0.65 (0.38–1.09)	0.61 (0.37–1.02)
Age group (years)						
18–24	Ref	Ref	Ref	Ref	Ref	Ref
25–39	0.62 (0.40–0.97)*	0.48 (0.25–0.91)*	0.75 (0.39–1.46)	2.44 (1.23–4.83)*	4.41 (1.02–19.10)*	2.08 (0.92–4.70)
40–54	0.81 (0.53–1.26)	0.71 (0.38–1.34)	0.87 (0.46–1.66)	3.39 (1.74–6.61)***	5.05 (1.18–21.50)*	3.21 (1.46–7.10)**
≥55	0.63 (0.40–0.98)*	0.45 (0.24–0.86)*	0.92 (0.46–1.85)	4.38 (2.21–8.66)***	8.66 (2.02–37.15)**	3.03 (1.32–6.95)**
Sex						
Male	Ref	Ref	Ref	Ref	Ref	Ref
Female	1.04 (0.87–1.25)	0.89 (0.70–1.12)	1.37 (1.02–1.83)*	1.01 (0.82–1.25)	1.17 (0.86–1.58)	0.85 (0.63–1.15)
Education						
Low	Ref	Ref	Ref	Ref	Ref	Ref
Moderate	1.02 (0.82–1.26)	0.82 (0.62–1.10)	1.37 (0.98–1.90)	0.89 (0.70–1.14)	1.13 (0.78–1.63)	0.74 (0.52–1.03)
High	0.93 (0.72–1.19)	0.77 (0.55–1.06)	1.23 (0.82–1.84)	0.90 (0.67–1.20)	1.13 (0.74–1.71)	0.73 (0.49–1.10)
Income						
Low	Ref	Ref	Ref	Ref	Ref	Ref
Medium	0.97 (0.76–1.24)	1.01 (0.74–1.36)	0.90 (0.59–1.38)	1.01 (0.77–1.34)	1.11 (0.76–1.61)	0.89 (0.58–1.36)
High	1.27 (0.99–1.62)	1.38 (1.00–1.91)*	1.17 (0.78–1.76)	0.80 (0.60–1.06)	0.75 (0.49–1.15)	0.77 (0.51–1.16)
No answer	0.88 (0.61–1.26)	0.97 (0.61–1.55)	0.75 (0.41–1.36)	1.27 (0.85–1.89)	1.07 (0.60–1.91)	1.42 (0.80–2.52)
Minority status						
Spoke non-English/non-white	Ref	Ref	Ref	Ref	Ref	Ref
Spoke English only/white	1.66 (1.17–2.35)**	1.41 (0.87–2.29)	2.06 (1.19–3.57)*	0.70 (0.48–1.03)	0.63 (0.34–1.16)	0.76 (0.46–1.26)
Heaviness of Smoking Index#	1.00 (0.93–1.06)	0.95 (0.87–1.04)	1.03 (0.93–1.14)	0.99 (0.92–1.07)	1.07 (0.95–1.21)	0.95 (0.86–1.06)
Wave of recruitment	1.01 (0.98–1.04)	0.99 (0.96–1.05)	1.01 (0.96–1.07)	0.97 (0.93–1.00)	0.96 (0.91–1.02)	0.97 (0.92–1.03)
Survey mode						
Web	Ref	Ref	Ref	Ref	Ref	Ref
Phone	1.21 (0.99–1.47)	0.97 (0.75–1.25)	1.64 (1.20–2.22)**	0.51 (0.41–0.64)***	0.72 (0.51–1.00)	0.37 (0.27–0.51)***

*AOR* = adjusted odds ratios; CI = confidence interval; estimates adjusted for variables in the table; EC = electronic cigarette; # coded as zero for ex-smokers.

*P < .05; **P < .01; ***P < .001.

Correlates of reported uncertainty about EC relative harmfulness were similar in the two countries, though in the opposite direction to those for “A lot less harmful” belief. “Don’t Know” responses were more likely to be given by respondents from Australia, who had not tried ECs, who had not seen EC ads on the internet, those from older age groups, and web survey respondents. In addition, those who had quit for more than a year were less likely to respond “Don’t Know” than current smokers.

## Discussion

The findings from this study show that smokers and ex-smokers in the United Kingdom are more likely than their counterparts in Australia to believe the almost certain fact that ECs are a lot less harmful than conventional cigarettes, but that there is more uncertainty about relative harmfulness in Australia. This result is consistent with the more permissive regulatory environment for ECs and the higher base rate of EC awareness and use observed in 2013 in the United Kingdom compared to Australia.^2^ The substantial expressed uncertainty about EC relative harmfulness observed in both countries is likely related to the ongoing public debate about the potential public health benefits and consequences of EC use.

The results also show that the characteristics of those who believe that ECs are a lot less harmful than conventional cigarettes are somewhat different in the two countries, suggesting that the type of regulatory environment is likely to affect who will hold such a view of EC relative risk profile. The mixed relationships between country and belief are hard to interpret. Some may reflect access to information, extent of exposure to them and the extent of disinformation.

Consistent with past studies,^2,10^ there was a strong positive association between having tried ECs and believing them to be a lot less harmful than conventional cigarettes, suggesting that those who hold favorable beliefs about EC relative harmfulness are more willing to try and/or continue to use ECs, and/or that trial prompts the search for information. By contrast, smokers from both countries who were uncertain about the relative harmfulness of ECs were less likely to have tried or to currently use ECs.

One key lesson from this study is that the regulatory framework for ECs adopted by a country can affect perceptions about the harmfulness of ECs relative to conventional cigarettes among smokers and ex-smokers. Jurisdictions with a more restrictive environment for ECs must recognize that misinformation and/or ignorance about EC relative risk in comparison to conventional cigarettes is likely substantial in their population, especially among subgroups who are not proactive in seeking out authoritative information. The high levels of public confusion and misinformation (64% and 42% of Australian and UK smokers, respectively, were misinformed in 2013) need to be remedied by evidence-based public education to ensure that smokers can make informed choices about the potential benefits of ECs to them, including an assessment of the risks. This is particularly important for smokers who are unable or unwilling to quit. It is unethical to resist informing current smokers of the potential benefits of switching to ECs by arguing that we do not know enough about the long-term risks of EC use, when we know almost certainly that ECs are considerably less harmful than conventional cigarettes.^9^

Several limitations warrant some discussion. First, because country and regulatory environment are confounded, some of the differences we attribute to the regulatory differences may be due to other country differences, including differences in publicly disseminated messages from health authorities. Also, less harmful belief about ECs was related to age and income, two sample characteristics that differed significantly between the two countries. However, the differences were in the direction that strengthens the findings, that is, the UK sample was less misinformed despite the fact that the respondents were older and less affluent. Given that the two countries are otherwise similar with regard to tobacco control policy and the fact that our models controlled for sociodemographic characteristics of participants, we consider the EC regulatory environment to be the most plausible explanation for the observed differences^2^ although contribution from dissimilar publicly disseminated messages about the potential risks of ECs from health authorities in the two countries could not be ruled out. Secondly, the cross-sectional findings here may not generalize over time as new product, new advertising standards and inaccurate media stories continue to appear. Thirdly, the observed country differences may reflect some methodological artefacts such as survey mode although this is likely to be minimal as this was controlled for in our models.

Consistent with the divergent regulatory environments affecting the sale and marketing of ECs in the two countries (ie, more restrictive in Australia and less restrictive in the United Kingdom) smokers and recent ex-smokers in the United Kingdom were more likely to believe ECs were less harmful relative to conventional cigarettes compared to smokers in Australia. The substantial expressed uncertainty observed in both countries is likely a result of ongoing public debate and scientific uncertainty of the potential benefits and the feared negative consequences of increased EC use at the societal level. Public education, based on science, may be helpful to remedy this situation, in particular, to reduce the substantial number of misinformed smokers in both countries.

## Supplementary Material

Supplementary Table 1 can be found online at http://www.ntr.oxfordjournals.org

## Funding

The ITC Four-Country Survey is supported by multiple grants including R01 CA100362 and P50 CA111236 (Roswell Park Transdisciplinary Tobacco Use Research Center) and also in part from grant P01 CA138389 and an ITC Pilot Study Grant (Medical University of South Carolina, Charleston, SC), all funded by the National Cancer Institute of the United States, Canadian Institutes of Health Research (79551, 115016), National Health and Medical Research Council of Australia (265903, 450110, APP1005922), and Cancer Research UK (C312/A3726 and C25586/A19540). SCH is a member of the UK Centre for Tobacco & Alcohol Studies, a UK Clinical Research Collaboration Public Health Research: Centre of Excellence whose work is supported by funding from the Medical Research Council, British Heart Foundation, Cancer Research UK, Economic and Social Research Council, and the National Institute for Health Research under the auspices of the UK Clinical Research Collaboration (MR/K023195/1).

## Declaration of Interests

*KMC has received grant funding from Pfizer, Inc. to study the impact of a hospital based tobacco cessation intervention and also has served as an expert witness in litigation filed against the tobacco industry. All other authors have no conflicts of interest to declare. All waves of the study have received ethical approval from the relevant research ethics committee at the Cancer Council Victoria (Australia) and the University of Strathclyde (United Kingdom).*
